# Stargardt Disease Presenting as Retinitis Pigmentosa: A Case of Monoallelic ABCA4-Associated Retinopathy

**DOI:** 10.7759/cureus.109345

**Published:** 2026-05-21

**Authors:** Kevin Santiago-Narváez, Ricardo A Murati Calderon, Natalio Izquierdo

**Affiliations:** 1 Department of Ophthalmology, School of Medicine, Medical Sciences Campus, University of Puerto Rico, San Juan, PRI

**Keywords:** abca4 mutation, inherited retinal disorders, p.asn1868ile variant, retinitis pigmentosa–like phenotype, stargardt disease

## Abstract

Stargardt disease is an inherited retinal disorder caused by mutations in the *ABCA4* gene and encompasses a wide range of phenotypes. Variability in age of onset, visual function, and retinal findings can make diagnosis challenging. We report a 48-year-old Hispanic female patient with a heterozygous *ABCA4* variant, c.5603A>T (p.Asn1868Ile), previously classified as benign. The patient had well-preserved best-corrected visual acuity of 20/40 in both eyes and normal color vision testing. Fundus examination revealed optic disc pallor, arteriolar attenuation, and peripheral bone spicule-like pigmentation in both eyes. Fundus autofluorescence demonstrated central hypoautofluorescence with surrounding hyperautofluorescence, suggestive of retinal pigment epithelium dysfunction. Spectral-domain optical coherence tomography showed preserved central macular contour with central subfield thickness (CST) of 298 µm in the right eye (OD) and 312 µm in the left eye (OS). Additionally, cubic volume was decreased in the OD at 9.1 mm³ and within normal limits in the OS at 9.6 mm³. Visual field testing demonstrated an annular pattern of visual field loss with significant mean deviation reduction (-17.17 dB in the OD and -16.12 dB in the OS). This adult-onset, RP-like presentation highlights the phenotypic variability of *ABCA4*-associated retinopathies and underscores the importance of cautious interpretation of monoallelic variants. The novelty of this case lies not in the mutation itself, but in the distinct clinical phenotype and diagnostic challenge it presents, reinforcing the need for careful genotype-phenotype correlation in inherited retinal disorders.

## Introduction

Karl Stargardt first described seven patients with a progressive macular degeneration that develops during early childhood or early adulthood [1]. Clinical manifestations in patients with the disease include gradual central vision deterioration, color vision abnormalities, and characteristic retinal findings, including yellow-white flecks in the posterior pole and the classic *dark choroid* sign on fluorescein angiography, which are highly suggestive of Stargardt disease [2,3]. The clinical spectrum in patients with Stargardt disease is heterogeneous. Maculopathy may range from early-onset severe to later-onset milder phenotypes. While classical Stargardt disease typically presents with central vision loss and macular atrophy in childhood or adolescence, certain *ABCA4* variants can result in atypical phenotypes, including cone-rod dystrophy or retinitis pigmentosa (RP)-like presentations characterized by peripheral retinal degeneration and visual field constriction. Stargardt disease affects up to one in 10,000 individuals, making it one of the most common inherited retinal disorders worldwide, after RP [2,3]. Clinical suspicion, multimodal retinal imaging, and confirmatory genetic testing are essential for diagnosis [3,4].

Stargardt disease is inherited as an autosomal recessive trait. Mutations in the *ABCA4* (ATP-binding cassette subfamily A member 4) gene are most commonly associated with this disease spectrum [3]. Over 1,200 mutations in *ABCA4* have been identified [5], contributing to significant clinical variability.

The most common manifestation is an inherited juvenile macular degeneration that primarily affects central vision [2,6,3]. Cone-rod dystrophy (CRD) may also develop, presenting with decreased visual acuity, increased light sensitivity, and impaired color vision [7]. Additionally, atypical forms such as RP, fundus flavimaculatus, and generalized choriocapillaris dystrophy have been described [8].

Variants in the *ABCA4* gene include missense, nonsense, and deep intronic mutations, each associated with different levels of residual protein function [9-11]. Genotype-phenotype correlation studies suggest that hypomorphic variants may demonstrate reduced penetrance and variable expressivity [9,10]. However, genetic findings should be interpreted cautiously, as panel-based testing may not detect all pathogenic variants, and clinical presentation remains central to diagnosis.

The *ABCA4* gene encodes a transporter protein found in photoreceptors that clears toxic retinoid byproducts from photoreceptor outer segment discs. Impairment of this process leads to lipofuscin accumulation in the retinal pigment epithelium (RPE), resulting in RPE atrophy, oxidative stress, photoreceptor degeneration, and progressive central vision loss. These changes typically manifest during the first or second decade of life [2,6].

Although no approved genetic therapy currently exists, advances in gene therapy, splicing correction, and pharmacologic approaches targeting lipofuscin accumulation are under investigation [12-14]. We report a patient with a known hypomorphic* ABCA4* variant whose clinical presentation was consistent with Stargardt disease.

## Case presentation

A 48-year-old Hispanic female patient with a medical history of pituitary microadenoma and fundus findings compatible with RP was referred to our clinic by a retina specialist for a genetic evaluation. The patient had no ocular complaints such as blurry vision, floaters, flashes, photophobia, or nyctalopia upon initial evaluation. Family history was notable for glaucoma in both parents. The patient was an active tobacco and alcohol user. Review of systems was completely unremarkable.

Upon comprehensive ophthalmic evaluation, the patient’s best corrected visual acuity was 20/40 in both eyes (OU). Intraocular pressures measured by applanation tonometry were 9 mmHg in the right eye (OD) and 10 mmHg in the left eye (OS). Gonioscopy was not performed, as the clinical suspicion at the time favored a retinal degenerative etiology rather than primary angle pathology. Her pupils were equal, round, and reactive to light, with no noticeable relative afferent pupillary defect in either eye. Extraocular movements were full and ortho in all directions of gaze. The Color Vision Hardy-Rand-Rittler test was intact in both eyes.

**Figure 1:**
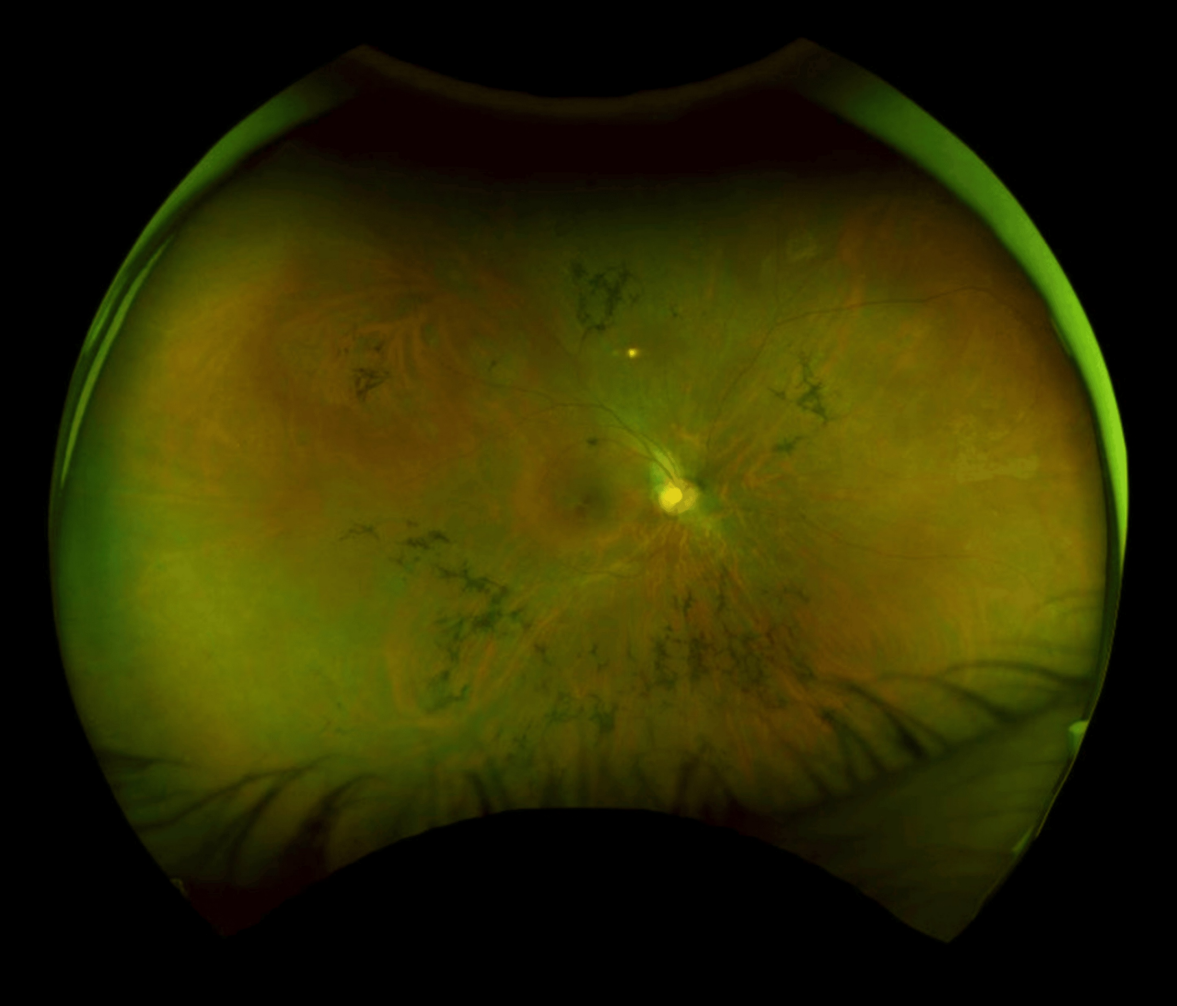
Ultra-widefield color fundus photography of the right eye demonstrating optic disc pallor, attenuation of retinal vessels, and mid-peripheral bone spicule-like pigmentation, consistent with a retinitis pigmentosa-like phenotype.

**Figure 2:**
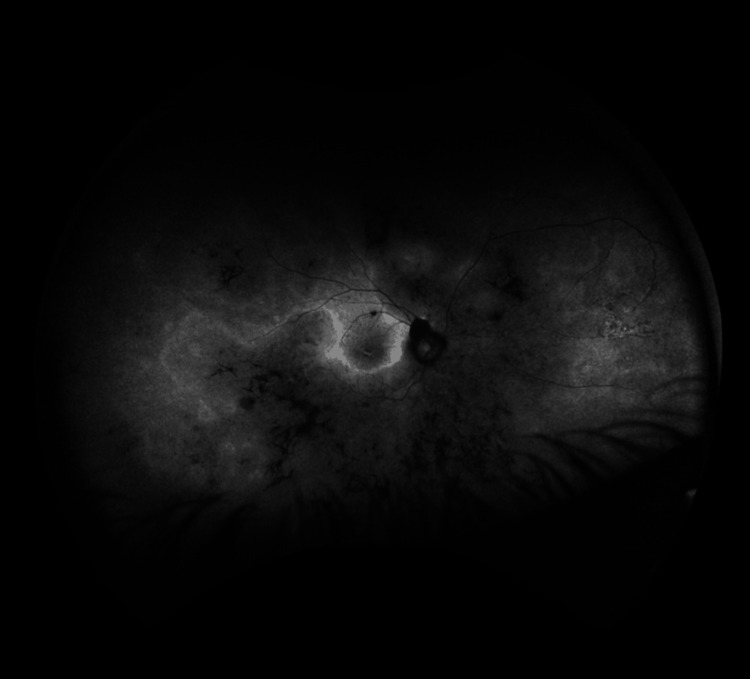
Fundus autofluorescence of the right eye demonstrating central hypoautofluorescence with surrounding areas of hyperautofluorescence, suggestive of retinal pigment epithelium dysfunction consistent with ABCA4-associated disease.

**Figure 3:**
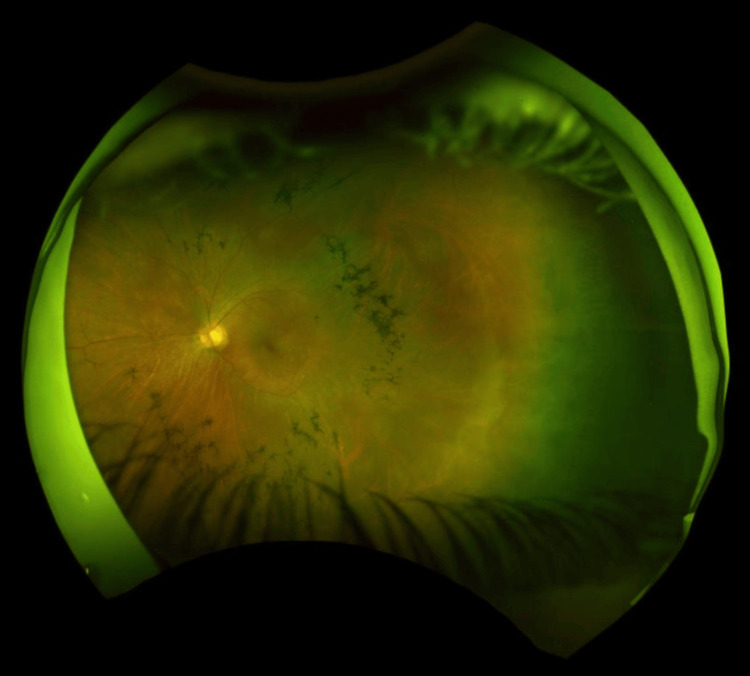
Ultra-widefield color fundus photography of the left eye demonstrating similar findings of optic disc pallor, vascular attenuation, and peripheral bone spicule-like pigmentation, supporting a bilateral process.

Anterior segment showed clear corneas, deep and quiet anterior chambers with no inflammation, and grade 1+ nuclear sclerosis in both lenses. Upon dilated fundus examination of the OD, there was optic disc pallor, attenuation of the retinal vasculature, and mid-peripheral bone spicule-like pigmentation (Figure 1). Fundus autofluorescence imaging revealed central hypoautofluorescence with surrounding areas of hyperautofluorescence, suggestive of RPE dysfunction (Figure 2). Similar funduscopic findings were observed in the OS, supporting a bilateral process (Figure 3).

Notably, the presence of mid-peripheral bone spicule-like pigmentation and arteriolar attenuation is characteristic of an RP-like phenotype, whereas the fundus autofluorescence pattern demonstrating central hypoautofluorescence with surrounding hyperautofluorescence is more consistent with *ABCA4*-associated retinopathy. This combination of findings highlights an overlapping phenotype that may mimic classic RP while retaining features suggestive of Stargardt disease.

**Figure 4:**
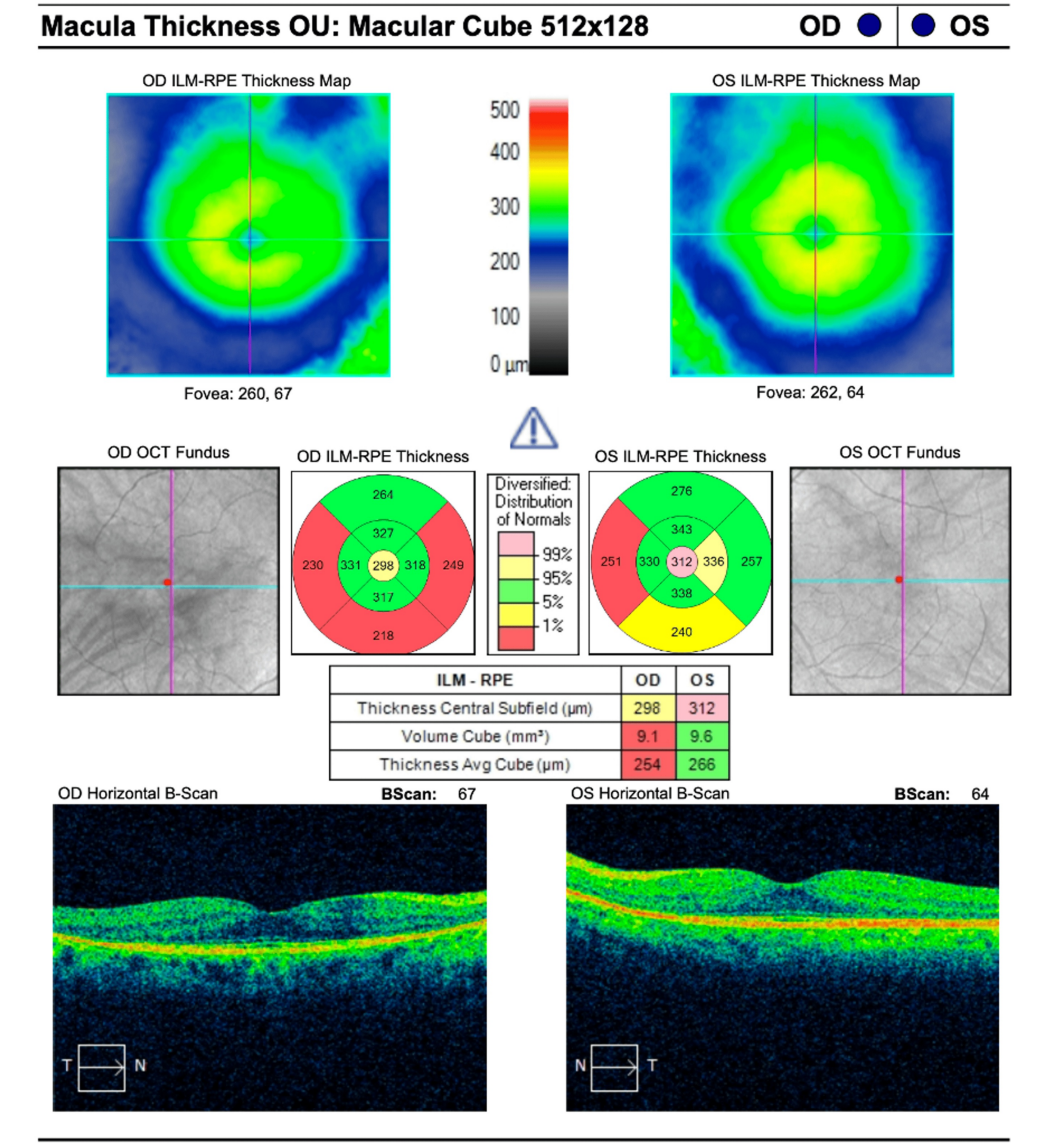
Macular spectral-domain OCT study showing a preserved macular contour in both eyes, with decreased macular thickness and volume in the right eye. Conventions: The normative color scale indicates values within normal limits (green: 5th-95th percentile), borderline (yellow: 1st-5th percentile), and outside normal limits (red: <1st percentile) compared with the device’s age-matched database. OCT, optical coherence tomography; ILM, inner limiting membrane; RPE, retinal pigment epithelium

Spectral-domain optical coherence tomography (OCT) of the macula (Cirrus; Carl Zeiss Meditec, Dublin, CA) showed a preserved macular contour in both eyes, without the significant macular atrophy typically seen in advanced Stargardt disease, with a central subfield thickness (CST) of 298 µm in the OD and 312 µm in the OS. Additionally, cubic volume was decreased in the OD, with a value of 9.1 mm³, and within normal range in the OS, with a value of 9.6 mm³ (Figure 4). 

**Figure 5:**
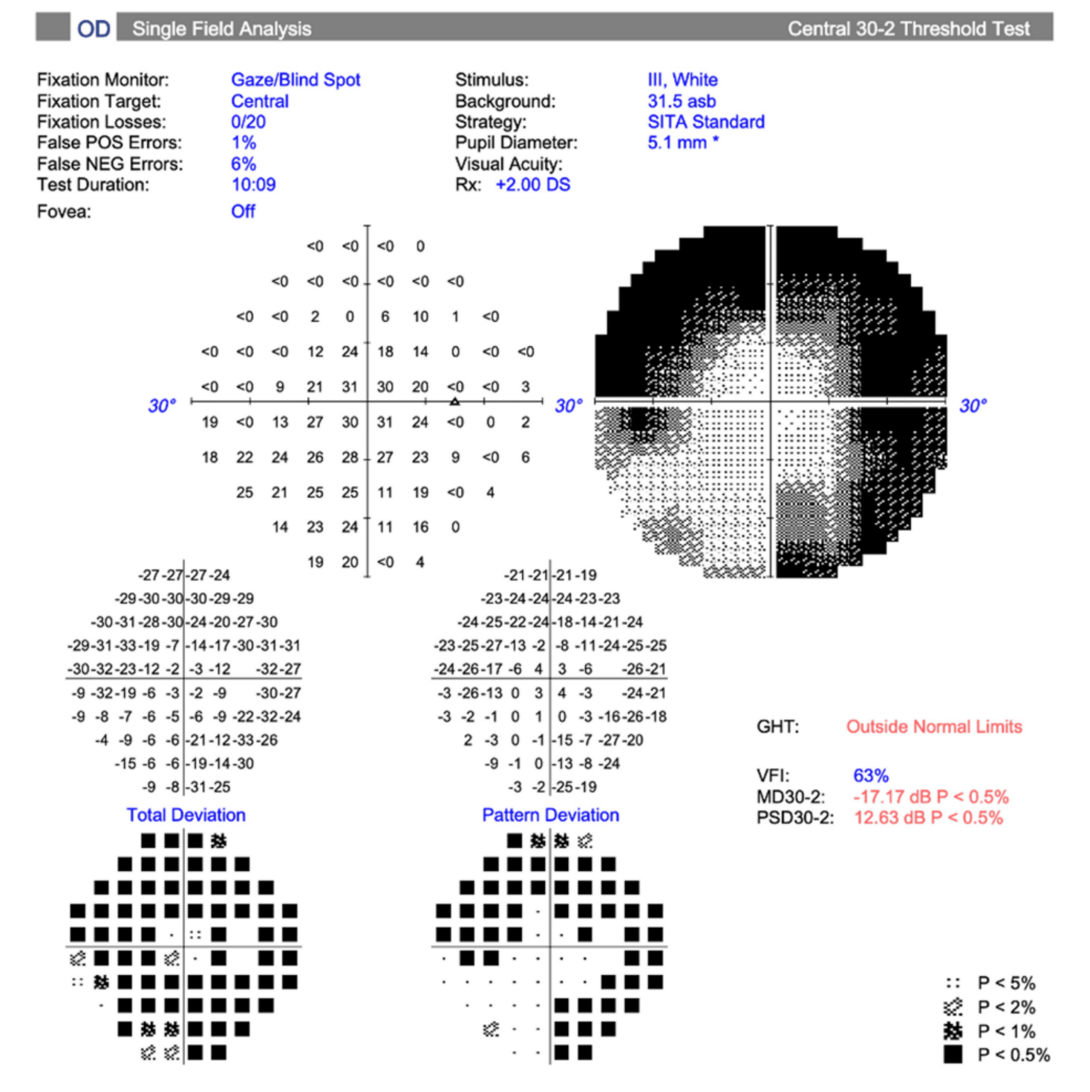
Visual field testing (30-2) of the right eye shows concentric peripheral visual field constriction with significantly decreased MD of -17.17 dB (p < 0.5%), PSD of 12.63 dB (p < 0.5%), and VFI of 63%. VFI, visual field index; MD, mean deviation; PSD, pattern standard deviation

**Figure 6:**
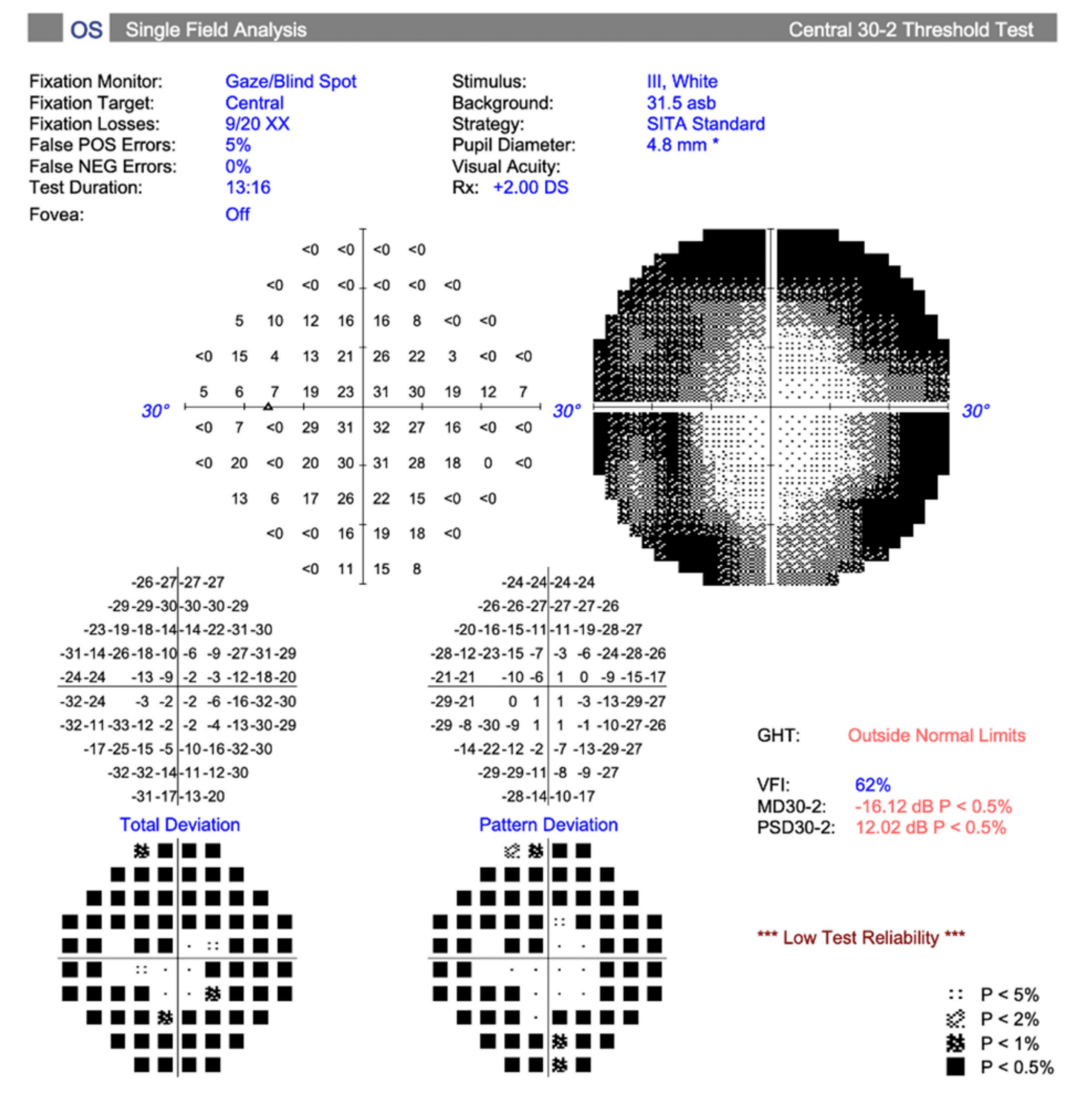
Visual field testing (30-2) of the left eye shows a ring scotoma with significantly decreased MD of -16.12 dB (p < 0.5%), PSD of 12.02 dB (p < 0.5%), and VFI of 62%. VFI, visual field index; MD, mean deviation; PSD, pattern standard deviation

As shown in Figures 5-6, Humphrey 30-2 Swedish Interactive Thresholding Algorithm (SITA) Standard visual field testing (Carl Zeiss Meditec) demonstrated concentric peripheral visual field constriction in the OD and a ring scotoma in the OS, consistent with an annular pattern of visual field loss, with glaucoma hemifield testing outside normal limits in both eyes. Both eyes had statistically significant mean deviation values of -17.17 dB in the OD and -16.12 dB in the OS.

Based on the characteristic ophthalmoscopic and multimodal imaging findings, a preliminary diagnosis of RP was reached. Genetic testing was conducted using a saliva sample and a next-generation sequencing (NGS) panel (Invitae Inherited Retinal Disorders Panel; Invitae Corporation, San Francisco, CA), which identified a heterozygous *ABCA4 *variant, c.5603A>T (p.Asn1868Ile), classified as benign. Despite the presumptive clinical diagnosis, the patient was counseled regarding the nature of the disease associated with the *ABCA4 *gene mutation and its progressive course.

Supportive management was initiated, focused on residual visual function. The patient was advised against using vitamin A supplements. The patient was referred for genetic counseling to discuss the implications of the findings, including the autosomal recessive inheritance pattern of *ABCA4*-associated disease and the limitations of panel-based testing. Further comprehensive genetic evaluation was recommended. The patient was advised to continue routine follow-up with a genetics specialist for surveillance of structural and functional progression. At the time of the last follow-up, her condition remained stable.

## Discussion

Previous studies have reported that patients with *ABCA4*-associated retinopathies display a wide range of phenotypes dependent on variant severity, residual protein function, and modifier effects. Early best-corrected visual acuity is often preserved despite measurable functional deficits, with progressive central vision loss as the disease advances [2,3]. Color vision abnormalities, frequently in the red-green or mixed axes, have also been documented in Stargardt disease and other *ABCA4*-related phenotypes [7]. In contrast, our patient’s best-corrected visual acuity remained 20/40 in both eyes, and color vision testing was normal, consistent with a later-onset phenotype that was initially diagnosed as retinitis pigmentosa. Overall, this case highlights the importance of integrating clinical, imaging, and genetic data when evaluating atypical retinal dystrophies.

Certain late-onset *ABCA4* phenotypes show preferential photoreceptor degeneration with preserved foveal architecture on optical coherence tomography [15], as observed in this case. Visual field findings in advanced disease often include paracentral scotomas [3], whereas our patient demonstrated dense central field loss (mean deviations −17.17 dB OD, −16.12 dB OS), potentially reflecting disease progression. Although normotensive glaucoma was considered in the differential diagnosis, the symmetric concentric peripheral field constriction across all quadrants and associated pigmentary retinal findings favored a retinal dystrophy over glaucomatous optic neuropathy. The absence of focal glaucomatous optic nerve cupping and the presence of characteristic pigmentary retinal changes further support a retinal dystrophy rather than primary glaucomatous optic neuropathy.

Riveiro-Alvarez et al. studied mutations in the *ABCA4* gene in Spanish populations [16]. Our patient’s next-generation sequencing study showed a heterozygous *ABCA4* variant, c.5603A>T (p.Asn1868Ile), which was classified as benign. It is important to note that a formal evaluation by a medical genetics specialist was not performed at the time of initial assessment, representing a limitation in the interpretation of the genetic findings. Despite the identification of only one detectable variant, the imaging and functional findings align closely with an *ABCA4*-associated phenotype. Expanded molecular testing, including whole-exome or whole-genome sequencing, may help identify undetected deep intronic or structural variants that are not captured by standard multigene panels. Given the known genetic heterogeneity of Stargardt disease, the possibility of a second undetected pathogenic allele or involvement of additional retinal dystrophy-associated genes cannot be excluded. Therefore, the genetic finding should not be interpreted as definitive causation in isolation from the clinical phenotype. Genotype-phenotype correlation studies indicate that more severe or null alleles correspond to earlier onset and more rapid progression, whereas hypomorphic alleles or deep-intronic variants often produce later-onset, slower disease, sometimes with foveal sparing [9,10,11]. It has also been noted that in many *ABCA4* phenotypes, only a single variant is detected, suggesting either a second undetected allele or that hypomorphic variants may have incomplete penetrance. For example, the p.Asn1868Ile variant may cause disease only in trans with a severe allele, and its penetrance has been estimated at less than 5% in some cohorts [5,17]. Finally, emerging studies emphasize the need for broader molecular testing and careful variant interpretation, especially as targeted therapies are under development [13,14]. Given Puerto Rico’s historical ties to Spain, shared ancestry could be a contributing factor, although this cannot be concluded from a single case.

In comparison with the published literature, our case fits the pattern described for hypomorphic *ABCA4* alleles in findings including: adult onset, preserved fovea with outer retinal damage, and slower disease progression [6,9,10]. The finding of a single heterozygous p.Asn1868Ile variant is also consistent with reports of low-penetrance alleles requiring additional unidentified molecular changes or modifier genes [5,17]. Although this variant was classified as benign, the patient’s phenotype raises the possibility that it may contribute to disease in the presence of additional, undetected genetic or environmental factors.

Unlike most classic juvenile *ABCA4* cases with biallelic severe variants, our patient’s presentation suggests a milder disease course, which correlates with late-onset cohorts [2,3]. This underscores the clinical implication that *ABCA4* retinopathy should be considered even in adult patients with atypical features and that a single hypomorphic allele does not exclude disease. Clinically, this enhances the case for expanded molecular diagnostics such as deep-intronic variant screening, copy-number analysis, or long-read sequencing and for genetic counseling that addresses non-classical onset and the possibility of unidentified second alleles [5,11].

Investigations into *ABCA4* function demonstrate that the protein acts to clear toxic retinoid byproducts from photoreceptor outer segment discs. Failure of this process results in lipofuscin accumulation, retinal pigment epithelium (RPE) atrophy, and secondary photoreceptor degeneration [6]. As targeted therapies evolve, adequately classifying our patients’ genotype and phenotype becomes increasingly important [12,13]. For this reason, the patient was advised not to use vitamin A supplements.

## Conclusions

This case describes an adult patient with RP-like fundus findings and a monoallelic* ABCA4* p.Asn1868Ile variant, highlighting the diagnostic complexity of* ABCA4*-associated retinopathies. Although variant p.Asn1868Ile is considered a hypomorphic, low-penetrance allele, our patient’s phenotype suggests that clinically significant disease may occur even when only a single variant is detected on standard panel testing. These findings reinforce the importance of careful genotype-phenotype correlation and consideration of expanded molecular testing when clinical suspicion for* ABCA4* disease remains high. 
